# Treatment of orofacial granulomatosis: a case report

**DOI:** 10.1186/s13256-017-1455-4

**Published:** 2017-10-25

**Authors:** Maaz B. Badshah, Saqib Walayat, Umair Ahmed, Sonu Dhillon, Sherri Yong, Sunanda Kane, Shoba Thievanayagam

**Affiliations:** 1 Department of Gastroenterology and Hepatology, University of Illinois Peoria Campus, OSF Saint Francis Medical Center, Peoria, IL 61637 USA; 2 Department of Internal Medicine, University of Illinois Peoria Campus, OSF Saint Francis Medical Center, Peoria, IL 61637 USA; 3 Department of Pahology, University of Illinois Peoria Campus, OSF Saint Francis Medical Center, Peoria, IL 61637 USA; 4 Mayo Clinic College of Medicine, Division of Gastroenterology & Hepatology, 200 First Street, SW, 19th Floor, Rochester, MN 55905 USA

**Keywords:** Cheilitis granulomatosa, Crohn’s disease, Infliximab, Orofacial granulomatosis

## Abstract

**Background:**

Orofacial granulomatosis is a relatively recent term coined by Wiesenfield *et al*. in 1985 to define granulomatous lesions of oral mucosa without intestinal involvement. When it presents in a triad encompassing facial nerve palsy, lip swelling, and fissured or furrowed tongue it is called Melkersson–Rosenthal syndrome while monosymptomatic or oligosymptomatic forms are referred to as granulomatous cheilitis. It is an uncommon clinicopathologic entity which is distinct from classic Crohn’s disease. The *NOD2* variant which is commonly associated with Crohn’s has not been shown to have any association with orofacial granulomatosis.

**Case presentation:**

We present a case of a 31-year-old white man who had painful swelling of the lip with oral ulcers and difficulty eating for 2 to 3 years. He was diagnosed as having granulomatous cheilitis based on characteristic biopsy findings. There was serologic evidence of Crohn’s disease with anti-*Saccharomyces cerevisiae* antibodies. However, he was not found to have any gastrointestinal involvement based on computed tomography enterography, and upper and lower endoscopies. He failed to respond to nonsteroidal anti-inflammatory drugs, steroids, and dapsone therapy but responded well to high doses of infliximab.

**Conclusions:**

Our case questions whether granulomatous cheilitis really exists or is it simply a variant of Crohn’s disease with only oral presentation. Our patient did not have symptoms of Crohn’s disease; moreover, endoscopic studies and computed tomography enterography were unremarkable for evidence of intestinal involvement. Our case is also the first reported case where high-dose infliximab alone has been used with sustained response for approximately 8 months. In conclusion, more research is needed to assess the underlying pathology as well as ideal treatment options for patients with orofacial granulomatosis. We propose that high-dose infliximab should be considered in patients who do not respond to traditional therapies.

## Background

Orofacial granulomatosis (OFG) is a relatively recent term coined by Wiesenfield *et al*. in 1985 to define granulomatous lesions of oral mucosa without intestinal involvement. It can present either as a triad involving facial nerve, lip swelling, and fissured or furrowed tongue, referred to as Melkersson–Rosenthal syndrome (MRS), or as its monosymptomatic or oligosymptomatic forms, referred to as granulomatous cheilitis (GC). GC is a persistent relapsing-remitting, idiopathic, nontender swelling of one or both lips [1, 2]. The etiology is unknown; however, relationships to Crohn’s disease (CD), sarcoidosis, and various infectious agents have been reported but none have been validated [3]. Because of the rarity of the condition there is no consensus on treatment options. Tumor necrosis factor-α (TNF-α) inhibitors have been reported to be of benefit in various reports. Here we report a case of OFG and our approach to treatment.

## Case presentation

A 31-year-old white man presented to our Gastroenterology clinic with complaints of painful swelling of the mouth with oral ulcers and difficulty eating. He endorsed that he has had ulcers and inflammation in the mouth for the past 2 to 3 years. He observed no bleeding or purulent exudate from the lesions. He was diagnosed with GC by biopsy revealing multiple non-caseating granulomas which were focally associated with multinucleated giant cells. Acid-fast bacilli and Gomori methenamine silver stains were negative for acid-fast and fungal organisms. No foreign material was seen with polarized light. He was treated with dapsone with minimal response. He had no history of abdominal surgeries, recent travel, or sick contacts. His vital signs were stable and he had no fever. A comprehensive physical examination revealed painful, right-sided lip, tongue, and facial swelling with tender sublingual ulcers along with an 8 mm shallow smooth-based ulcer just below his last molar with some surrounding edema and erythema. His saliva was normal with no yellowing. There was no lymphadenopathy palpated. The rest of his examination was unremarkable. Laboratory work revealed a normal hemoglobin, platelet count, and CRP. Mandibular X-rays and an ultrasound showed no fractures or abscesses in the affected area. An magnetic resonance imaging (MRI) of his head and neck suggested a soft tissue lesion, along the buccal aspect of his right mandible and maxilla. A positron emission tomography (PET) scan showed nonspecific fairly symmetric metabolic activity in bilateral cervical lymph nodes.

Further laboratory evaluation included: a negative QuantiFERON tuberculosis (TB) test; negative heavy metal testing for arsenic, lead, and mercury; normal angiotensin-converting enzyme (ACE levels), negative celiac panel, and negative *Helicobacter pylori* serology. Inflammatory bowel disease (IBD) serology showed positive anti-*Saccharomyces cerevisiae* antibodies (ASCA). Our patient denied any gastrointestinal (GI) symptoms but underwent endoscopy and colonoscopy to rule out possibility of CD. There were two small superficial ulcerations seen in his mid-gastric body and erythema and erosions in the antrum and duodenum. Biopsies revealed nonspecific chronic active gastritis and duodenitis. Colonoscopy with intubation of the cecum was unremarkable. Computed tomography (CT) enterography was normal. He was started on high-dose proton pump inhibitor (PPI) therapy for chronic gastritis and high-dose infliximab (10 mg/kg every 4 weeks) given his symptoms to which he had excellent response. Our patient has been on Remicade (infliximab) for almost 8 months with sustained response.

## Discussion

Cheilitis granulomatosa is a persistent, idiopathic, nontender swelling of the lips [1]. It is considered a manifestation of OFG, a non-necrotizing granulomatous inflammatory disorder characterized by persistent or recurrent soft tissue enlargement, oral ulceration, and a variety of orofacial features, in the absence of systemic granulomatous disorders such as CD or sarcoidosis [2, 4]. The prevalence of oral lesions in CD varies from 5 to 50%. Oral lesions may be the first presenting sign in patients with CD in 5 to 10% of the cases or rarely the only manifestation [5–7].

Our patient did not have any GI symptoms and his endoscopic biopsies did not show any granulomatosis. Traditionally GC does not respond to biologics, but our patient had an excellent response. There are only a handful of cases reporting the use of immunomodulators for treatment of OFG without intestinal involvement (Table 1) [8–13]. Gaya *et al*. reported the case of a patient with cheilitis who responded initially to 5 mg/kg infliximab with good response within 7 days but was subsequently lost to follow-up [8]. The patient re-presented later with recurrence and was treated with adalimumab with only partial response [8]. Barry *et al*. initially tried doses of 3 mg/kg with response initially followed by relapse; therefore, they increased the dose to 5 mg/kg with sustained response [9]. Kakimoto *et al*. reported using 5 mg/kg at 0, 2, and 6 weeks with good response initially followed by infusion reaction, the patient was therefore switched to adalimumab for successful treatment of MRS [11]. Ruiz Villaverde and Sánchez Cano reported using 80 mg of adalimumab initially followed by 40 mg every 2 weeks for a total of 6 months with sustained response and no relapse post treatment in a patient with GC [12]. Their patient had initially failed to respond to prednisone (oral and intralesional), topical tacrolimus, orally administered roxithromycin, and clofazimine [12]. Stein *et al*. also reported using adalimumab 80 mg followed by 40 mg every 3 weeks along with 20 mg/day prednisone with resolution of symptoms in their patient with MRS, they later tapered therapy to 40 mg every 4 weeks for a total duration of therapy of 21 months [13]. Their patient remained symptom free 5 years post treatment on follow-up [13]. Their patient had failed to respond to methotrexate, prednisone, and azathioprine [13]. Peitsch *et al*. also have reported the use of infliximab 5 mg/kg at 2, 6, and 10 weeks followed by maintenance therapy every 8 weeks in their patient with OFG [14]. Their patient had failed to respond to methylprednisolone, dapsone, hydroxychloroquine, and sulfasalazine [14]. Caution should be used before starting TNF-α and underlying infection should be ruled out. Also, screening for mycobacterial diseases should be carried out before starting these drugs.

**Table 1:** List of various treatment modalities reported by other authors for treatment of orofacial granulomatosis

Authors and Reference	Primary disease	Treatment approach	Dose	Response
Gaya *et al*. [8]	Orofacial granulomatosis	Infliximab followed by adalimumab	5 mg/kg	Responded initially, lost to follow-up, partial response later
Barry *et al*. [9]	Granulomatous cheilitis	Infliximab	3 mg/kg initially followed by 5 mg/kg	Initial relapse at 3 mg/kg but sustained response at 5 mg/kg
Kakimoto *et al*. [11]	Melkersson–Rosenthal Syndrome	Infliximab followed by adalimumab	5 mg/kg	Adverse reaction to infliximab but sustained response to adalimumab
Ruiz Villaverde and Sánchez Cano [12]	Granulomatous cheilitis	Adalimumab	80 mg followed by 40 mg	Sustained response post 6 month treatment
Stein *et al*. [13]	Melkersson–Rosenthal Syndrome	Adalimumab	80 mg followed by 40 mg	Sustained response post 21 month treatment
Peitsch *et al*. [14]	Orofacial granulomatosis	Infliximab	5 mg/kg	Sustained response

This case raises the question whether granulomatous cheilitis really exists or is it simply a variant of CD limited to the oral cavity [15]. We believe that OFG is a distinct clinical entity from CD, as is supported by genetic studies which have shown that the common *NOD2* CD risk variant showed no association with OFG alone or OFG plus CD [16]. Worssae *et al*. also found no evidence of association between CD and MRS [3]. Moreover, the excellent clinical response of our patient suggests that aggressive biologic therapy, when conventional treatments fail, is a reasonable strategy for OFG with or without the presence of underlying CD. While there are case reports where infliximab has been used in combination with various therapies like azathioprine, to the best of our knowledge our case report is the first to report the use of high-dose infliximab (10 mg/kg every 4 weeks) as the sole treatment for orofacial granulomatosis with good response.

## Conclusions

In conclusion, we report that OFG is a distinct clinical entity from CD as reported in our case where there was no evidence of underlying CD despite presence of ASCA. Moreover, we believe that high-dose infliximab may be an alternative treatment option in patients who do not respond initially with other treatment regimens; however, more studies need to be done before definitive conclusions can be made.

## Abbreviations

ASCA: Anti-*Saccharomyces cerevisiae* antibodies
CD: Crohn’s disease
GC: Granulomatous cheilitis
GI: Gastrointestinal
MRS: Melkersson–Rosenthal syndrome
OFG: Orofacial granulomatosis
TNF-α: Tumor necrosis factor-α
